# Synthesis and Antimicrobial Studies of Some Novel Bis-[1,3,4]thiadiazole and Bis-thiazole Pendant to Thieno[2,3-*b*]thiophene Moiety

**DOI:** 10.3390/ijms13033661

**Published:** 2012-03-19

**Authors:** Nabila Abdelshafy Kheder, Yahia Nasser Mabkhot

**Affiliations:** 1 Department of Chemistry, Faculty of Science, Cairo University, Giza 12613, Egypt; E-Mail: nabila_abdelshafy@yahoo.com; 2 On leave to Department of Pharmaceutical Chemistry, Faculty of Pharmacy, King Khalid University, P. O. Box 418 Abha 61431, Saudi Arabia; 3 Department of Chemistry, Faculty of Science, King Saud University, P. O. Box 2455 Riyadh 11451, Saudi Arabia

**Keywords:** thieno[2,3-*b*]thiophene, nucleophilic addition, hydrazonoyl halides, bis-thiadiazoles, bis-thiazoles, antimicrobial activity

## Abstract

The synthetic utility of 3,3′-(3,4-dimethylthieno[2,3-*b*]thiophene-2,5-diyl)bis (3-oxopropanenitrile) (**1**) in the synthesis of some novel bis-[1,3,4-thiadiazole] **6a–g** and bis-thiazole **10** and **13** derivatives with thieno[2,3-b]thiophene moiety is reported. Antimicrobial evaluation of some selected examples from the synthesized products was carried out and showed promising results.

## 1. Introduction

Thiophene compounds are well known to exhibit various biological and medicinal activities such as BACE1 inhibitors [1], antitubercular [2], anti-depressant [3], anti-inflammatory [4], anti-HIV PR inhibitors [5], and anti-breast cancer activities [6]. In addition, thienothiophenes have potential applications in a wide variety of optical and electronic systems [7–9]. Furthermore, 1,3,4-thiadiazoles were recently reported as highly anti-inflammatory [10,11], and anticonvulsant agents [10,12]. Also, thiazoles and their derivatives found application in drug development for the treatment of allergies [13], hypertension [14], inflammation [15], schizophrenia [16], bacterial [17] and HIV infections [18]. Encouraged by all these findings and in continuation of our ongoing research program investigating the utilization of compound **1** as versatile and useful building blocks for the synthesis of a wide variety of bis-heterocycles systems [19,20], we report in the present work an efficient and rapid method for the synthesis of a series of thienothiophene pendant to thiadiazole or thiazole moieties.

## 2. Results and Discussion

**Scheme 1:**
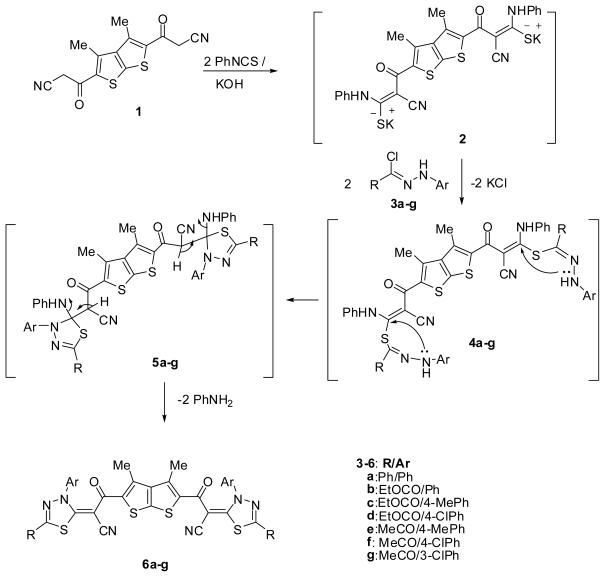
Synthesis of bis-[1,3,4]-thiadiazole structures **6a–g**.

The nucleophilic addition of thieno[2,3-*b*]thiophene **1** [19] to phenyl isothiocyanate in DMF, in the presence of potassium hydroxide, afforded the corresponding potassium salt **2**. Heterocyclisation of the intermediate **2** with hydrazonoyl chlorides **3a** [21] or **3b–d** [22] or **3e–g** [23] furnished in each case, one isolable product (as tested by TLC). The reaction products were identified as bis-[1,3,4]-thiadiazole structures **6a–g** (Scheme 1).

The structure of the products **6a–g** was determined from spectroscopic as well as elemental analytical data. Thus, compound **6a**, taken as a typical example, showed absorption bands at 1674 and 2199 cm^−1^ corresponding to C=O and C ≡ N groups, respectively. Its ^1^H NMR spectrum revealed the absence of CH_2_ protons of compound **1** and showed signals at δ 2.49 due to CH_3_ protons, in addition to an aromatic multiplet in the region δ 7.57–7.97. The aforementioned results indicate that the reaction proceeds via *S*-alkylation [24] to give *S*-alkylated intermediate **4** which cyclized *in situ* under the employed reaction conditions to give intermediate **5**. Elimination of two aniline molecules from **5** gave the desired product **6** (Scheme 1).

**Scheme 2:**
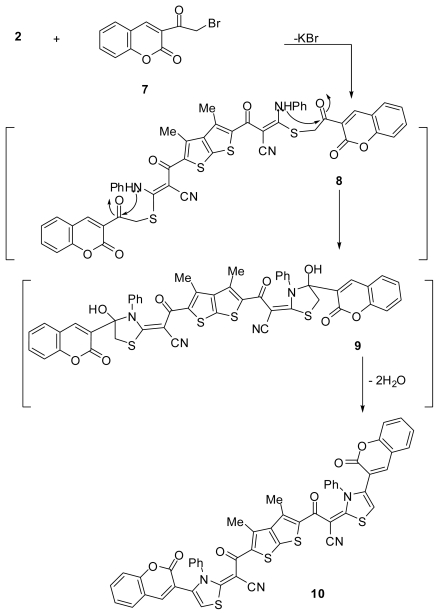
Synthesis of 3,3′-(3,4-dimethylthieno[2,3-*b*]thiophene-2,5-diyl)bis(3-oxo-2-(4- (2-oxo-2*H*-chromen-3-yl)-3-phenylthiazol-2(3*H*)-ylidene)propanenitrile (**10**).

Next, the reactivity of the potassium salt **2** towards 3-(2-bromoacetyl)-2*H*-chromen-2-one (**7**) [25,26] was also investigated. Thus, treatment of potassium salt **2** with compound **7** gave one product that was identified as 3,3′-(3,4-dimethylthieno[2,3-*b*]thiophene-2,5-diyl)bis(3-oxo-2-(4-(2-oxo-2*H*-chromen-3-yl)-3-phenylthiazol-2(3*H*)-ylidene)propanenitrile) (**10**) as shown in Scheme 2. The reaction proceeds via nucleophilic displacement of bromide to give *S*-alkylated intermediate **8**, followed by nucleophilic addition of (PhNH) group to carbonyl group of chromen-2-one ring to give the respective intermediate **9**. Dehydration of the latter intermediate gave bis-thiazole derivative **10** as the final product. The IR spectrum of the isolated product showed absorption bands at 2195, 1647 and 1724 cm^−1^ due to nitrile function and carbonyl groups, respectively. Its ^1^H NMR spectrum showed singlet signal at *δ* 2.49 ppm due to methyl protons, in addition to aromatic multiplets in the region *δ* 7.02–8.6 ppm.

**Scheme 3:**
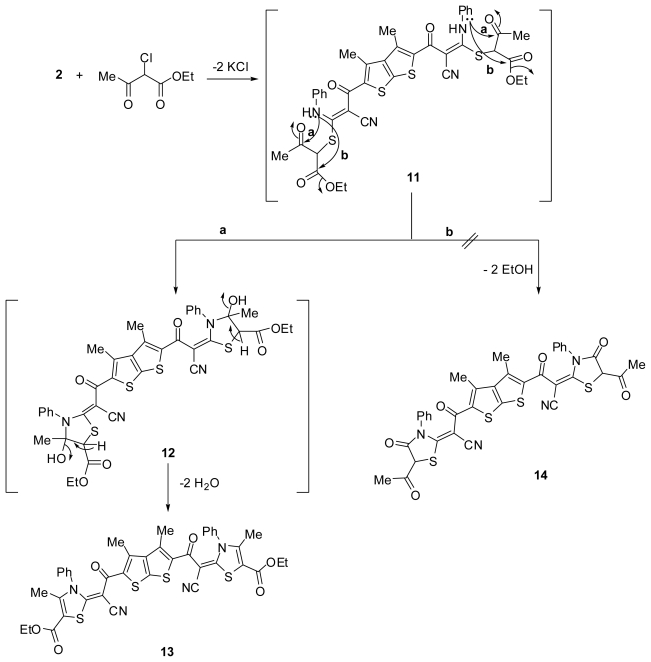
Synthesis of diethyl 2,2′-(2,2′-(3,4-dimethylthieno[2,3-*b*]thiophene-2,5- diyl)bis(1-cyano-2-oxoethan-2-yl-1-ylidene))bis(4-methyl-3-phenyl-2,3-dihydrothiazole-5- carboxylate) (**13**).

Similarly, treatment of the potassium salt **2** with ethyl 2-chloro-3-oxobutanoate afforded diethyl 2,2′-(2,2′-(3,4-dimethylthieno[2,3-*b*]thiophene-2,5-diyl)bis(1-cyano-2-oxoethan-2-yl-1-ylidene))bis(4- methyl-3-phenyl-2,3-dihydrothiazole-5-carboxylate) (**13**) as outlined in Scheme 3. The bis-thiazole structure **13** was confirmed from its elemental analyses and spectral data. The IR spectrum of compound **13** revealed absorption bands at 2206, 1713 and 1643 cm^−1^ due to nitrile function and two carbonyl groups, respectively. Its ^1^H-NMR spectrum showed a triplet signal at δ 1.30 (*J* = 7.2 Hz) due to CH_3_ protons, two singlet signal at δ 2.24 and 2.49 characteristics for two methyl protons, a quartet signal at δ 4.32 (*J* = 7.2 Hz) due to CH_2_ protons, in addition to an aromatic multiplet in the region δ 7.62. A proposed mechanism for the formation of the bis-thiazole structure **13** is depicted in Scheme 3. The foregoing spectral data supported the proposed structure **13** and ruled out the other bis-thiazole structure **14** (Scheme 3).

## 3. Experimental Section

All melting points were measured on a Gallenkamp melting point apparatus (Weiss-Gallenkamp, London, UK). The infrared spectra were recorded in potassium bromide disks on a pye Unicam SP 3300 and Shimadzu FT IR 8101 PC infrared spectrophotometers (Pye Unicam Ltd. Cambridge, England and Shimadzu, Tokyo, Japan, respectively). The NMR spectra were recorded on a BRUKER VX-500 NMR spectrometer (Varian, Palo Alto, CA, USA). ^1^H spectra were run at 500 MHz in deuterated dimethyl sulfoxide (DMSO-*d_6_*). Chemical shifts were related to that of the solvent. Elemental analyses were carried out at the Micro-analytical Center of Cairo University, Giza, Egypt. The biological evaluation of the products **6a–g** and **10** were carried out in the Medical Mycology Laboratory of the Regional Center for Mycology and Biotechnology of Al-Azhar University, Cairo, Egypt. Thieno[2,3-b]thiophene **1** [19], and hydrazonoyl chlorides **3a** [21], **3b–d** [22], **3e–g** [23], and 3-(2-bromoacetyl)-2*H*-chromen-2-one (**7**) [25,26] were prepared following the literature procedure.

### Reactions of Compound **1** with Hydrazonoyl Halides **3a** or **3b-d** or **3e-g** or 3-(2-bromoacetyl)-2Hchromen- 2-one (**7**)

#### General Procedure

To a stirred solution of potassium hydroxide (0.11 g, 2 mmol) in 20 mL DMF was added compound **1** (0.302 g, 1 mmol). After stirring for 30 min, phenyl isothiocyanate (0.27 g, 2 mmol) was added to the resulting mixture. Stirring was continued for 6 h, and then the appropriate hydrazonoyl chlorides **3a–g** (2 mmol) or 3-(2-bromoacetyl)-2*H*-chromen-2-one (**7**) (0.534 g, 2 mmol) or ethyl 2-chloro-3-oxobutanoate (0.329 g, 2 mmol) was added portion-wise over a period of 30 min. After the addition was complete, the reaction mixture was stirred for additional 12 h, during which the hydrazonoyl chloride or 3-(2-bromoacetyl)-2*H*-chromen-2-one went into solution and a yellow product precipitated. The solid product was filtered off, washed with EtOH and dried, Recrystallization from DMF/EtOH (3:1) afforded the corresponding bis-[1,3,4]thiadiazole derivatives **6a–g** or bis-thiazole derivatives **10** or **13**, respectively.

##### 3,3′-(3,4-Dimethylthieno[2,3-b]thiophene-2,5-diyl)bis(2-(3,5-diphenyl-1,3,4-thiadiazol-2(3H)- ylidene)-3-oxopropanenitrile) (**6a**)

Yield (61%), m.p. 276 °C; IR (KBr) ν_max_: 2905 (aliphatic CH), 2199 (C ≡ N), 1674 (C=O) cm^−1; 1^H-NMR (DMSO-d_6_): δ2.49 (s, 6H, 2CH_3_), 7.57–7.97 (m, 20H, ArH). MS m/z (%): 775 (M^+^, 0.16), 774 (0.14), 471 (46.73), 304 (4.13), 77 (70.79). Anal. Calcd for C_42_H_26_N_6_O_2_S_4_ (774.95): C, 65.09; H, 3.38; N, 10.84. Found: C, 65.01; H, 3.45; N, 10.90%.

##### Diethyl 5,5′-(2,2′-(3,4-dimethylthieno[2,3-b]thiophene-2,5-diyl)bis(1-cyano-2-oxoethan-2-yl-1- ylidene))bis(4-phenyl-4,5-dihydro-1,3,4-thiadiazole-2-carboxylate)(**6b**)

Yield (52%), m.p. > 300 °C; IR (KBr) ν_max_: 2982 (aliphatic CH), 2199 (C≡N), 1744 and 1674 (2C=O) cm^−1; 1^H-NMR (DMSO-d_6_): *δ* 1.33 (s, 6H, 2CH_3_, *J* = 6.9 Hz), 2.49 (s, 6H, 2CH_3_), 4.44 (q, 4H, 2CH_2_, *J* = 6.9 Hz),7.53–7.92 (m, 10H, ArH). MS m/z (%): 767 (M^+^, 1.57), 167 (19.92), 149 (36.71), 77 (7.77). Anal. Calcd for C*_36_*H*_26_*N*_6_*O*_6_*S*_4_* (766.89): C, 56.38; H, 3.42; N, 10.96. Found: C, 56.30; H, 3.36; N, 10.88%.

##### Diethyl 5,5′-(2,2′-(3,4-dimethylthieno[2,3-b]thiophene-2,5-diyl)bis(1-cyano-2-oxoethan-2-yl-1- ylidene))bis(4-p-tolyl-4,5-dihydro-1,3,4-thiadiazole-2-carboxylate) (**6c**)

Yield (66%), m.p. > 300 °C; IR (KBr) ν_max_: 2986 (aliphatic CH), 2203 (C≡N), 1747 and 1674 (2C=O) cm^−1; 1^H-NMR (DMSO-d_6_): *δ* 1.35 (s, 6H, 2CH_3_, *J* = 7.0 Hz), 2.42 (s, 6H, 2CH_3_), 2.52 (s, 6H, 2CH_3_),4.46 (q, 4H, 2CH_2_, *J* = 7.0 Hz),7.41 (d, 4H, *J* = 8.0 Hz), 7.62 (d, 4H, *J*= 8.0 Hz). MS m/z (%): 793 (3.44), 222 (4.85), 221 (4.55), 167 (9.11), 91 (50.33), 77 (51.22). Anal. Calcd for C_38_H_30_N_6_O_6_S_4_ (794.94): C, 57.41; H, 3.80; N, 10.57. Found: C, 57.52; H, 3.88; N, 10.66 %.

##### Diethyl 5,5′-(2,2′-(3,4-dimethylthieno[2,3-b]thiophene-2,5-diyl)bis(1-cyano-2-oxoethan-2-yl-1- ylidene))bis(4-(4-chlorophenyl)-4,5-dihydro-1,3,4-thiadiazole-2-carboxylate)(**6d**)

Yield (53%), m.p. > 300 °C; IR (KBr) ν_max_: 2986 (aliphatic CH), 2206 (C≡N), 1744 and 1674 (2C=O) cm^−1; 1^H-NMR (DMSO-d_6_): δ1.37 (s, 6H, 2CH_3_, *J* = 7.0 Hz), 2.52 (s, 6H, 2CH_3_), 4.47 (q, 4H, 2CH_2_, *J* = 7.0 Hz),7.73 (d, 4H, *J*= 10.0 Hz), 7.84 (d, 4H, *J* = 10.0 Hz). MS m/z (%): 835 (M^+^, 2.81), 334 (6.05), 168 (8.37), 112 (6.37), 111 (23.38), 77 (39.48). Anal. Calcd for C_36_H_24_Cl_2_N_6_O_6_S (835.78): C, 51.73; H, 2.89; N, 10.06. Found: C, 51.67; H, 2.79; N, 10.12%.

##### 3,3′-(3,4-Dimethylthieno[2,3-b]thiophene-2,5-diyl)bis(2-(5-acetyl-3-p-tolyl-1,3,4-thiadiazol-2(3H)- ylidene)-3-oxopropanenitrile)(**6e**)

Yield (52%), m.p. 240 °C; IR (KBr) ν_max_: 2199 (C≡N), 1690 and 1674 (2C=O) cm^−1; 1^H-NMR (DMSO-d_6_): *δ* 2.29 (s, 6H, 2CH_3_), 2.45 (s, 6H, 2CH_3_), 2.50 (s, 6H, 2CH_3_), 7.22 (d, 4H, *J* = 8.5 Hz), 7.33 (d, 4H, *J* = 8.5 Hz). MS m/z (%): 732 (0.04), 647 (0.06), 221 (2.03), 166 (1.33), 106 (100.0), 91, (58.18), 77 (84.54). Anal. Calcd for C_36_H_26_N_6_O_4_S_4_ (734.89): C, 58.84; H, 3.57; N, 11.44. Found: C, 58.77; H, 3.49; N, 11.38%.

##### 3,3′-(3,4-Dimethylthieno[2,3-b]thiophene-2,5-diyl)bis(2-(5-acetyl-3-(4-chlorophenyl)-1,3,4- thiadiazol-2(3H)-ylidene)-3-oxopropanenitrile)(**6f**)

Yield (49%), m.p. 295 °C; IR (KBr) ν_max_: 2199 (C≡N), 1693 and 1655 (2C=O) cm^−1; 1^H-NMR (DMSO-d_6_): *δ* 2.41 (s, 6H, 2CH_3_), 2.52 (s, 6H, 2CH_3_), 7.72 (d, 4H, *J* = 8.8 Hz), 7.84 (d, 4H, *J* = 8.8 Hz). MS m/z (%): 776 (3.02), 500 (3.36), 471 (9.6), 304 (3.99), 276 (6.27), 166 (10.71), 112 (6.32), 111 (16.73). Anal. Calcd for C_34_H_20_Cl_2_N_6_O_4_S_4_ (775.73): C, 52.64; H, 2.60; N, 10.83. Found: C, 52.58; H, 2.54; N, 10.77%.

##### 3,3′-(3,4-Dimethylthieno[2,3-b]thiophene-2,5-diyl)bis(2-(5-acetyl-3-(3-chlorophenyl)-1,3,4- thiadiazol-2(3H)-ylidene)-3-oxopropanenitrile)(**6g**)

Yield (49%), m.p. > 300 °C; IR (KBr) ν_max_: 2199 (C≡N), 1690 and 1647 (2C=O) cm^−1; 1^H-NMR (DMSO-d_6_): *δ*1.89 (s, 6H, 2CH_3_), 2.49 (s, 6H, 2CH_3_), 6.97–8.00 (m, 8H, ArH). MS m/z (%): 771 (3.28), 304 (6.34), 166 (22.08), 112 (13.36), 111 (18.98). Anal. Calcd for C_34_H_20_Cl_2_N_6_O_4_S_4_ (775.73): C, 52.64; H, 2.60; N, 10.83. Found: C, 52.55; H, 2.52; N, 10.74%.

##### 3,3′-(3,4-Dimethylthieno[2,3-b]thiophene-2,5-diyl)bis(3-oxo-2-(4-(2-oxo-2H-chromen-3-yl)-3- phenylthiazol-2(3H)-ylidene)propanenitrile) (**10**)

Yield (68%), m.p. > 300 °C; IR (KBr) ν_max_: 2195 ((C≡N)), 1724 and 1647 (2C=O) cm^−1; 1^H-NMR (DMSO-d_6_): *δ* 2.49 (s, 6H, 2CH_3_), 7.02–8.6 (m, 22H, ArH). MS m/z (%): 909 (2.45), 166 (2.75), 145 (4.05), 77 (15.41). Anal. Calcd for C_50_H_28_N_4_O_6_S_4_ (909.04): C, 66.06; H, 3.10; N, 6.16. Found: C, 66.15; H, 3.21; N, 6.25%.

##### Diethyl 2,2′-(2,2′-(3,4-dimethylthieno[2,3-b]thiophene-2,5-diyl)bis(1-cyano-2-oxoethan-2-yl-1- ylidene))bis(4-methyl-3-phenyl-2,3-dihydrothiazole-5-carboxylate) (**13**)

Yield (44%), m.p. 278–280 °C; IR (KBr) ν_max_: 2986 (aliphatic CH), 2206 ((C≡N)), 1713 and 1643 (2C=O) cm^−1; 1^H-NMR (DMSO-d_6_): *δ* 1.30 (t, 6H, 2CH_3_, *J* = 7.2 Hz), 2.24 (s, 6H, 2CH_3_), 2.49 (s, 6H, 2CH_3_), 4.32 (q, 4H, 2CH_2_, *J* = 7.2 Hz),7.62 (s, 10H, ArH). Anal. Calcd for C_40_H_32_N_4_O_6_S_4_ (792.97): C, 60.59; H, 4.07; N, 7.07. Found: C, 60.48; H, 4.16; N, 7.15%.

### 3.1. Antimicrobial Evaluation

The newly synthesized target compounds (**6a–g** and **10**) were evaluated for their *in vitro* antibacterial activity against *Staphylococcus aureus* (SA) and *Bacillis subtilis* (BS) as examples of Gram-positive bacteria and *Pseudomonas aeruginosa* (PA) and *Escherichia coli* (EC) as examples of Gram-negative bacteria. They were also evaluated for their *in vitro* antifungal potential against *Aspergillus fumigatus* (AF), *Geotrichum candidum* (GC), *Candida albicans* (CA) and *Syncephalastrum racemosum* (SR) fungal strains. The organisms were tested against the activity of solutions of concentrations (5 μg/mL) and using inhibition zone diameter (IZD) in mm as criterion for the antimicrobial activity (agar diffusion method). The fungicides *Itraconazole*, *Clotrimazole* and the bactericides *Penicillin G, Streptomycin* were used as references to evaluate the potency of the tested compounds under the same conditions. The results are depicted in Table 1.

The results depicted in Table 1 revealed that most of the tested compounds displayed variable inhibitory effects on the growth of the tested Gram-positive bacteria and Gram-negative bacteria strains and also against fungal strains. In general, most of the tested compounds revealed better activity against the Gram-positive bacteria rather than the Gram-negative bacteria: Compounds **6a**, **6c–d** and **10** exhibited almost no activity against *Syncephalastrum racemosum* and *Pseudomonas aeruginosa*; Compounds **6b** and **6e–g** exhibited almost no activity against *Candida albicans*. Compounds **6e** and **6g** exhibited almost no activity against *Pseudomonas aeruginosa*; Compounds **6d**, **6f** and **10** showed comparatively good activity against all the bacterial and fungal strains. The good activity of **6d** and **6f** is attributed to the presence of pharmacologically active 4-chlorophenyl at position 4 of the thiadiazole ring.

## 4. Conclusions

In conclusion, the reactivity of diethyl 3,3′-(3,4-dimethylthieno[2,3-*b*]thiophene-2,5-diyl)bis (3-oxopropanenitrile) (**1**) was investigated as a versatile and readily accessible building block for the synthesis of new bis-heterocycles incorporating thieno[2,3-*b*]thiophene moiety of biological and pharmaceutical importance.

## Figures and Tables

**Table 1:** Antibacterial and antifungal activities of the synthesized compounds (**6a–g**) and **10**.

Sample/Tested Organism	6a	6b	6c	6d	6e	6f	6g	10	Standard	

*Fungi*									Itraconazole	Clotrimazole
***Aspergillus fumigatus*(AF)**	11.7 ± 0.2	15.4 ± 0.09	13.3 ± 0.2	16.4 ± 0.3	9.3 ± 0.2	17.4 ± 0.08	12.2 ± 0.09	14.3 ± 0.2	28.5 ± 0.05	26 ± 0.1
***Geotrichum candidum*(GC)**	13.5 ± 0.1	14.9 ± 0.05	14.4 ± 0.1	18.1 ± 0.08	11.4 ± 0.1	18.3 ± 0.3	14.4 ± 0.03	16.7 ± 0.08	27.1 ± 0.06	23.1 ± 0.03
***Candida albicans*(CA)**	10.4 ± 0.08	NA	10.2 ± 0.09	13.7 ± 0.05	NA	NA	NA	11.9 ± 0.1	26.1 ± 0.02	18.3 ± 0.01
***Syncephalastrum racemosum*(SR)**	NA	12.1 ± 0.08	NA	NA	8.2 ± 0.09	14.2 ± 0.08	9.2 ± 0.08	NA	22.3 ± 0.09	20.5 ± 0.02
***Gram Positive Bacteria***									**Penicillin G**	**Streptomycin**
***Staphylococcus aureus*(SA)**	11.2 ± 0.1	17.9 ± 0.05	11.3 ± 0.05	15.4 ± 0.5	9.4 ± 0.05	18.9 ± 0.01	13.8 ± 0.1	13.4 ± 0.3	29.4 ± 0.08	25.1 ± 0.08
***Bacillis subtilis*(BS)**	13.7 ± 0.07	16.1 ± 0.01	9.0 ± 0.08	18.4 ± 0.1	10.6 ± 0.08	20.9 ± 0.03	16.6 ± 0.03	14.7 ± 0.09	32.5 ± 0.06	29.1 ± 0.04
***Gram Negative Bacteria***									**Penicillin G**	**Streptomycin**
***Pseudomonas aeruginosa*(PA)**	NA	10.1 ± 0.01	NA	NA	NA	12.1 ± 0.01	NA	NA	28.3 ± 0.05	24.3 ± 0.08
***Escherichia coli*(EC)**	8.3 ± 0.09	14.5 ± 0.2	10.1 ± 0.07	13.7 ± 0.05	7.4 ± 0.07	15.2 ± 0.5	9.5 ± 0.2	10.9 ± 0.2	33.5 ± 0.7	25.6 ± 0.04

NA: No activity, data are expressed in the form of mean ± SD. Mean zone of inhibition in mm ± Standard deviation beyond well diameter (6 mm) produced on a range of environmental and clinically pathogenic microorganisms using (5 mg/mL) concentration of tested samples.

## References

[1] Giordanetto F., Karlsson O., Lindberg J., Larsson L.O., Linusson A., Evertsson E., Morgan D.G.A., Inghardt T. (2007). Discovery of cyclopentane- and cyclohexane-trans-1,3-diamines as potent melanin-concentrating hormone receptor 1 antagonists. Bioorg. Med. Chem. Lett.

[2] Parai M.K., Panda G., Chaturvedi V., Manju Y.K., Sinha S. (2008). Thiophene containing triarylmethanes as antitubercular agents. Bioorg. Med. Chem. Lett.

[3] Wardakhan W.W., Abdel-Salam O.M.E., Elmegeed G.A. (2008). Screening for antidepressant, Sedative and analgesic activities of novel fused thiophene derivatives. Acta Pharm.

[4] Kumar P.R., Raju S., Goud P.S., Sailaja M., Sarma M.R., Reddy G.O., Kumar M.P., Reddy V.V., Suresh T., Hegde P. (2004). Synthesis and biological evaluation of thiophene [3,2-*b*] pyrrole derivatives as potential anti-inflammatory agents. Bioorg. Med. Chem.

[5] Bonini C., Chiummiento L., Bonis M.D., Funicello M., Lupattelli P., Suanno G., Berti F., Campaner P. (2005). Synthesis, biological activity and modelling studies of two novel anti HIV PR inhibitors with a thiophene containing hydroxyethylamino core. Tetrahedron.

[6] Brault L., Migianu E., Néguesque A., Battaglia E., Bagrel D., Kirsch G. (2005). New thiophene analogues of kenpaullone: Synthesis and biological evaluation in breast cancer cells. Eur. J. Med. Chem.

[7] Litvinov V.P. (2005). The latest achievements in thienothiophene chemistry. Russ. Chem. Rev.

[8] Gather M.C., Heeny M., Zhang W., Whitehead K.S., Bradley D.D.C., McCulloch I., Campbell A.J. (2008). An alignable fluorene thienothiophene copolymer with deep-blue electrolumenescent emission at 410 nm. Chem. Commun.

[9] He M., Li J., Sorensen M.L., Zhang F., Hancock R.R., Fong H.H., Pozdin V.A., Smilgies D., Malliaras G.G. (2009). Alkylsubstituted thienothiophene semiconducting materials: Structure property relationships. J. Am. Chem. Soc.

[10] Dawood K.M., Abdel-Gawad H., Ragab E.A., Ellithey M., Mohamed H.A. (2006). Synthesis, anticonvulsant, and anti-inflammatory evaluation of some new benzotriazole and benzofuran-based heterocycles. Bioorg. Med. Chem.

[11] Schenone S., Bruno O., Ranise A., Bondavalli F., Filippelli W., Falcone G., Giordano L., Vitelli M.R. (2001). 3-Arylsulphonyl-5-arylamino-1,3,4-thiadiazol-2(3H)ones as anti-inflammatory and analgesic agents. Bioorg. Med. Chem.

[12] Ilies M.A., Masereel B., Rolin S., Scozzafava A., Câmpeanu G., Cîmpeanu V., Supuran C.T. (2004). Carbonic anhydrase inhibitors: Aromatic and heterocyclic sulfonamides incorporating adamantyl moieties with strong anticonvulsant activity. Bioorg. Med. Chem.

[13] Hargrave K.D., Hess F.K., Oliver J.T. (1983). *N*-(4-Substituted-thiazolyl)oxamic acid derivatives, new series of potent, orally active antiallergy agents. J. Med. Chem.

[14] Patt W.C., Hamilton H.W., Taylor M.D., Ryan M.J., Taylor D.G., Connolly C.J.C., Doherty A.M., Klutchko S.R., Sircar I., Steinbaugh B.A. (1992). Structure-activity relationships of a series of 2-amino-4-thiazole-containing renin inhibitors. J. Med. Chem..

[15] Sharma P.K., Sawnhney S.N., Gupta A., Singh G.B., Bani S. (1998). Synthesis and antiinflammatory activity of some 3-(2-thiazolyl)-1,2-benzisothiazoles. Indian J. Chem.

[16] Jean J.C., Wise L.D., Caprathe B.W., Tecle H., Bergmeier S., Humblet C.C., Heffner T.G., Meltzner L.T., Pugsley T.A. (1990). 4-(1,2,5,6-Tetrahydro-1-alkyl-3-pyridinyl)-2-thiazolamines: A novel class of compounds with central dopamine agonist properties. J. Med. Chem.

[17] Tsuji K., Ishikawa H. (1994). Synthesis and anti-pseudomonal activity of new 2-isocephems with a dihydroxypyridone moiety at C-7. Bioorg. Med. Chem. Lett.

[18] Bell F.W., Cantrell A.S., Hogberg M., Jaskunas S.R., Johansson N.G., Jordon C.L., Kinnick M.D., Lind P., Morin J.M., Noreen R. (1995). Synthesis and basic structure-activity relationship studies of PETT analogs. J. Med. Chem.

[19] Mabkhoot Y.N. (2009). Synthesis and analysis of some bis-heterocyclic compounds containing sulphur. Molecules.

[20] Mabkhoot Y.N, Kheder N.A., Al-Majid A.M. (2010). Facile and convenient synthesis of new thieno[2,3-*b*]thiophene derivatives. Molecules.

[21] Wolkoff P. (1975). A new method of preparing hydrazonyl halides. Can. J. Chem.

[22] Shawali A.S., Eweiss N.F., Hassaneen H.M., Al-gharib M.S. (1975). Synthesis and rearrangement of ethyl aryloxyglyoxalate arylhydrazones. Bull. Chem. Soc. Jpn.

[23] Eweiss N.F., Osman A. (1980). Synthesis of heterocycles. Part II. New routes to acetylthiadiazolines and alkylazothiazoles. J. Heterocycl. Chem.

[24] Geies A.A., Kamal-Eldeen A.M., Abdelhafez A.A., Gaber A.M. (1991). Synthesis of some thiazolo(3,2-a) pyrimidines. Phosphor. Sulfur Silicon Relat. Elem.

[25] Koelsch C.F. (1950). Bromination of acetocoumarin. J. Am. Chem. Soc.

[26] Czerney P., Hartman H. (1983). 3-α-bromacetyl-coumarines as synthones for heterocyclic substituted coumarines. J. Prakt. Chem.

